# Clinical Characteristics and Spatial Transcriptome Analysis of Non–Small Cell Lung Cancers Exhibiting Early Alectinib Resistance: A Retrospective OLCSG Study

**DOI:** 10.1158/2767-9764.CRC-25-0545

**Published:** 2026-02-06

**Authors:** Tadahiro Kuribayashi, Go Makimoto, Kadoaki Ohashi, Shuta Tomida, Hirofumi Inoue, Toshihide Yokoyama, Shoichi Kuyama, Yuka Kato, Kenichiro Kudo, Naokatsu Horita, Hiroe Kayatani, Masaaki Inoue, Keisuke Sugimoto, Kiichiro Ninomiya, Yoshinobu Maeda, Yosuke Togashi, Katsuyuki Hotta

**Affiliations:** 1 Department of Hematology, Oncology and Respiratory Medicine, Okayama University Graduate School of Medicine, Dentistry and Pharmaceutical Sciences, Okayama, Japan.; 2 Department of Respiratory Medicine, Okayama University Hospital, Okayama, Japan.; 3 Center for Comprehensive Genomic Medicine, Okayama University Hospital, Okayama, Japan.; 4 Department of Respiratory Medicine, Ohara Healthcare Foundation, Kurashiki Central Hospital, Kurashiki, Japan.; 5 Department of Respiratory Medicine, NHO Iwakuni Clinical Center, Iwakuni, Japan.; 6 Department of Thoracic Oncology and Medicine, National Hospital Organization, Shikoku Cancer Center, Matsuyama, Japan.; 7 Department of Respiratory Medicine, National Hospital Organization Okayama Medical Center, Okayama, Japan.; 8 Department of Respiratory Medicine, Kure Kyosai Hospital, Kure, Japan.; 9 Department of Respiratory Medicine, Japanese Red Cross Okayama Hospital, Okayama, Japan.; 10 Department of Chest Surgery, Shimonoseki City Hospital, Shimonoseki, Japan.; 11 Department of Respiratory Medicine, Japanese Red Cross Kobe Hospital, Kobe, Japan.; 12 Department of Tumor Microenvironment, Faculty of Medicine, Dentistry and Pharmaceutical Sciences, Okayama University, Okayama, Japan.; 13 Center for Innovative Clinical Medicine, Okayama University Hospital, Okayama, Japan.

## Abstract

Some *anaplastic lymphoma kinase* (*ALK*) gene rearrangement–positive lung cancers show early resistance, within 3 months, to alectinib. This study investigated the clinical and molecular characteristics of these patients. We analyzed patients with unresectable stage III/IV disease without indications for radical radiotherapy and recurrent *ALK*-positive lung cancer who received alectinib as the primary ALK tyrosine kinase inhibitor between 2013 and 2021 at nine hospitals. In total, 103 patients were included. The median age was 65 years; 44 were male and 22 had brain metastases. The median progression-free survival and overall survival (OS) were 28.7 and 80.6 months. Nineteen patients treated for ≤3 months and 84 treated for >3 months were categorized into the early resistance and responder groups, respectively. The early resistance group had significantly shorter OS (8.4 months vs. not estimable, *P* < 0.001) and was significantly more likely to have brain metastases (42% vs. 17%, *P* = 0.027). They also showed elevated inflammatory markers, including neutrophil-to-lymphocyte ratio (NLR). Univariate analysis identified brain metastases and high NLR as significant predictors of early resistance. Spatial transcriptome analysis and immunohistochemical staining revealed upregulation of annexin A1 (ANXA1), a calcium-dependent phospholipid-binding protein involved in inflammation and cancer progression, in the early resistance group. Interleukin 6 stimulation, prompted by elevated inflammatory markers, increased ANXA1 expression and reduced alectinib sensitivity. Knockdown of ANXA1 improved alectinib sensitivity in alectinib-resistant cells. In conclusion, brain metastases and high NLR are associated with early resistance. ANXA1 may play an important role in mediating early resistance. New treatment options for the early resistance group are required.

**Significance:**

Some *ALK*-positive lung cancers show early resistance to alectinib. This study found brain metastases and elevated NLR associated with early resistance. Spatial transcriptomics and functional analysis suggested involvement of ANXA1. These results reveal clinical and molecular biomarkers that may guide treatment strategies for early resistance to alectinib.

## Introduction

Lung cancer has the highest mortality rate worldwide (1). *Anaplastic lymphoma kinase* (*ALK*) gene rearrangement–positive non–small cell lung cancer (NSCLC) accounts for 4% to 5% of all NSCLC cases (2). ALK tyrosine kinase inhibitors (ALK-TKI), such as crizotinib, alectinib, brigatinib, ceritinib, and lorlatinib, have improved survival in patients with *ALK*-positive NSCLC (3). Alectinib, a second-generation ALK-TKI, provides longer progression-free survival (PFS) than crizotinib in *ALK*-positive NSCLC (4, 5). However, certain patients show disease progression within 3 months of starting alectinib (4, 5). In this study, we define PFS ≤3 months as “early resistance” based on major clinical trials demonstrating that fewer than 10% of patients progressed within this interval when treated with alectinib (4, 5). These patients have significantly worse outcomes and could potentially benefit from alternative first-line therapies or a more intensive initial management, highlighting the importance of early identification.

We previously reported that the causes of early resistance to alectinib are high tumor mutation burden and heterogeneous tumor evolution (6). Other factors include secondary mutations or amplification of the ALK tyrosine kinase domain, activation of bypass signaling pathways, drug efflux pumps, and phylogenetic changes (7, 8). In addition, clinical factors that have been reported to contribute to the efficacy of ALK-TKIs include inflammation markers such as the neutrophil-to-lymphocyte ratio (NLR), platelet-to-lymphocyte ratio (PLR), and systemic immune-inflammation index (SII; refs. 9–11). However, reliable clinical and molecular biomarkers that can predict early alectinib resistance remain to be established.

In recent years, technological progress has greatly facilitated the identification and development of cancer biomarkers (12). Among these, spatial transcriptomic analysis has emerged as a valuable tool for visualizing and analyzing tumor heterogeneity and the spatial distribution of biomarkers (13). GeoMx digital spatial profiling (DSP) enables simultaneous, highly multiplex spatial profiling of proteins or RNA in formalin-fixed paraffin-embedded (FFPE) tissues and has recently been used to identify prognostic and effect predictors at the protein and RNA levels (14). GeoMx has been used to identify spatially localized expression patterns of treatment-relevant biomarkers within tumor regions in lung cancer, demonstrating its utility for uncovering resistance-associated molecular mechanisms that may be difficult to detect using bulk analyses (15, 16). However, spatial transcriptomic analysis has not yet been applied to investigate early resistance to alectinib in *ALK*-positive NSCLC.

Therefore, we aimed to identify the clinical and molecular characteristics of patients with *ALK*-positive NSCLC who are early resistant to alectinib by integrating clinical data analysis and spatial transcriptomic profiling.

## Materials and Methods

### Patients and study design

This retrospective observational study included patients with unresectable stage III/IV disease without indications for radical radiotherapy and recurrent *ALK*-positive lung cancer who received alectinib as the primary ALK-TKI at nine collaborating hospitals of Okayama Lung Cancer Study Group between January 2013 and December 2021. The data for this study were collected in July 2023. *ALK* rearrangements were assessed using a test approved by the Pharmaceuticals and Medical Devices Agency of Japan, with immunohistochemistry (IHC) and/or fluorescence *in situ* hybridization. This study was approved by the Ethics Committee of the participating institutions [no. 2207-023; Institutional Review Board (IRB) of Okayama University Hospital] and was conducted in accordance with the Declaration of Helsinki. Given the retrospective nature of the study, the IRB waived the requirement for written informed consent. Instead, oral informed consent was obtained from patients who attended the hospital, and for patients who were unable to attend the hospital for any reason, an opt-out approach was approved by the IRB. Information regarding the opt-out process was made publicly available on the website of each participating institution. We categorized patients with a PFS >3 months into the responder group and those with a PFS ≤3 months into the early resistance group. To examine the characteristics of patients with early resistance to alectinib, we compared the early resistance and responder groups.

### Data collection

The clinical data of the patients were collected from the electronic medical records of each hospital. The data included age, sex, stage, Eastern Cooperative Oncology Group performance status (PS), histology, smoking history, brain and liver metastases, adverse events, and laboratory findings. The NLR, PLR, SII, and prognostic nutritional index (PNI) were calculated as follows: NLR = neutrophil count (/μL)/lymphocyte count (/μL), PLR = platelet count (/μL)/lymphocyte count (/μL), SII = platelet count (/μL) × neutrophil count (/μL)/lymphocyte count (/μL), and PNI = 10 × albumin (g/dL) + 0.005 × lymphocyte count (/μL).

### Outcomes

The primary outcome was overall survival (OS) in the early resistance and responder groups. Secondary outcomes were PFS in the two groups and the clinical and molecular factors correlated with early resistance to alectinib. PFS was calculated from the date of alectinib initiation to the date of disease progression or death from any cause. OS was calculated from the date of alectinib initiation to the date of death or last follow-up. Treatment effectiveness was evaluated according to the Response Evaluation Criteria in Solid Tumors version 1.1 (17).

### DSP experiments and data preprocessing

We followed published experimental methods (14) and the manufacturer’s instructions for the GeoMx Human Whole Transcriptome Atlas Human RNA for Illumina Systems (GMX-RNA-NGSHuWTA-4, NanoString). Briefly, serially sectioned FFPE sections (5 μm) were prepared under the IRB-approved protocol to generate consecutive sections that were processed for hematoxylin and eosin stain and whole transcriptome atlas (WTA), respectively. For WTA, slides were baked at 60°C for 30 minutes, deparaffinized, rehydrated, and washed using a Leica BOND Rx system (Leica Biosystems, RRID: SCR_025548). Targets were retrieved using the BOND Epitope Retrieval Solution 2 (Leica Biosystems, AR0087). The tissues were incubated in 1 ng/mL proteinase K for 15 minutes at 37°C and washed with PBS. Next, tissues were incubated overnight at 37°C with hybridization solution (WTA Probe Mix targeting 18,269 genes and Buffer R with DEPC-treated H_2_O). After incubation, tissues were washed twice with Stringent Wash Solution (4X SSC and 100% formamide) at 37°C for 25 minutes each, washed twice in 2X SSC for 2 minutes each, incubated in blocking buffer W for 30 minutes at room temperature in a humidity chamber, and then counterstained with morphologic markers for 1 hour at room temperature. The morphologic markers used were 1:10 SYTO13, 1:40 anti-panCK Alexa Fluor 532, 1:40 anti-CD45 Alexa Fluor 594 (NanoString GeoMx Solid Tumor Kit, cat. #GMX-RNA-MORPH-HST-12), and 1:500 anti-CD68 Alexa Fluor 647 (Santa Cruz, SC-20060, RRID: AB_3073741) in blocking buffer W (NanoString). These four morphologic markers allowed delineation of the nuclear, epithelial, immune, and macrophage compartments. Immunofluorescence images, region of interest (ROI) selection, segmentation into marker-specific areas of illumination (AOI), and spatially indexed barcode cleavage and collection were performed using a GeoMx DSP instrument (NanoString). Library preparation was performed according to the manufacturer’s instructions, and PCR amplification was performed to add Illumina adapter sequences and unique dual-sample indices. A minimum sequencing depth of 100 reads per square micron of the illumination area was achieved by sequencing all WTA AOIs on a NovaSeq platform (Illumina).

FASTQ files for DSP were aggregated into count matrices as described previously (14). Briefly, deduplicated sequencing counts were calculated based on unique molecular identifier and molecular target tag sequences. Targets that consistently fell below the limit of quantitation were removed, and the datasets were normalized using upper quartile (Q3) normalization.

### IHC

IHC was performed as described previously (18). FFPE tissue blocks were cut into 5-μm slices and placed on glass slides. Slides were deparaffinized using Hemo-De (FALMA, cat. #CS-1001-4) for 15 minutes and graded alcohol for 6 minutes. The slides were incubated in pure water containing Tris-EDTA Antigen Retrieval Buffer (Proteintech, cat. #PR30002) or Sodium Citrate Antigen Retrieval Buffer (Proteintech, cat. #PR30001) for 10 minutes in a pressure chamber at 95°C (Pascal S2800; Dako, cat. #S2800). The sections were then incubated with 0.3% hydrogen peroxide in methanol for 5 minutes to inactivate endogenous peroxidase. Slides were rinsed with TBS containing 0.1% Tween 20 and blocked with goat serum for 60 minutes at room temperature. The sections were incubated with anti–annexin A1 (ANXA1) antibody (Cell Signaling Technology, D5V2T, RRID: AB_2799031; 1:400), anti–claudin 4 (CLDN4) antibody (Abcam, EPRR17575, RRID: AB_2732879; 1:4,000), anti–interleukin 6 (IL-6) antibody (Abcam, ab6672, RRID: AB_2127460; 1:200), phospho-STAT3 (Cell Signaling Technology, D3A7, RRID: AB_2491009; 1:200), and anti–lipocalin 2 (LCN2) antibody (Cell Signaling Technology, D4M8L, RRID: AB_2799257; 1:3,000) overnight at 4°C. Sections were then incubated with a secondary antibody (Dako; cat. #K4003) for 20 minutes at room temperature and then reacted with 3,3-diaminobenzidine (Dako; cat. #K3468). Finally, the sections were counterstained with hematoxylin (MUTO Pure Chemicals, cat. #30002).

### Cell lines

We investigated the expression of ANXA1, CLDN4, and LCN2 in *EML4–ALK* fusion*–*positive cell lines. H3122 (*EML4–ALK* fusion; RRID: CVCL_5160) cells were kindly provided by Dr. William Pao (Vanderbilt University). The ABC-14 (*EML4–ALK* fusion; RRID: CVCL_A1EF), ABC-17 (*EML4–ALK* fusion; RRID: CVCL_A1EG), ABC-19 (*EML4–ALK* fusion), and ABC-23 (*EML4–ALK* fusion) cell lines were established in our laboratory from the patients who exhibited early resistance to alectinib (6, 19). Cell line identity was confirmed by short tandem repeat polymorphism analysis. All cell lines were cultured with a maximum of 20 passages. The cell lines were verified as *Mycoplasma*-free before starting the experiments [e-Myco Mycoplasma PCR Detection Kit (version 2.0), iNtRON Biotechnology, Inc.; cat. #25235]. All cell lines were cultured in RPMI-1640 medium (Nacalai Tesque; cat. #30264-85) supplemented with 10% heat-inactivated fetal bovine serum and 1% penicillin/streptomycin; cells were maintained at 37°C in a 5% CO_2_ humidified atmosphere.

### siRNA transfection

To assess the relationship between ANXA1 expression and the sensitivity to alectinib, siRNA transfection was performed. Transfection conditions for siRNA-mediated gene knockdown were optimized using ANXA1 siRNAs (QIAGEN, SI03063648 and SI03067568; cat. #1027416) and Lipofectamine RNAiMAX Transfection Reagent (Thermo Fisher Scientific; cat. #13778030) in six-well plates (300,000 cells/well). Two predesigned gene-specific siRNAs were tested for each candidate gene, along with negative controls (QIAGEN; cat. #10272780). Gene silencing efficiency was evaluated 72 hours after transfection by Western blot (WB) analysis.

### Cell proliferation assay

Growth inhibition was measured using a modified MTT assay. Briefly, cells were seeded in 96-well plates at densities of 2,000 to 5,000 cells/well (H3122: 2,000 cells/well, ABC-14: 5,000 cells/well). H3122 cells were grown in the presence or absence of human recombinant IL-6 (R&D Systems, cat. #206-IL) for 24 hours and then treated with alectinib (Selleck) at the indicated concentrations in the presence or absence of IL-6 for 72 hours. For rescue experiments, H3122 cells stimulated with IL-6 for 24 hours were treated with alectinib at the indicated concentrations with or without ruxolitinib (1 μmol/L; Selleck) for 72 hours. ABC-14 cells were treated with alectinib (0.1 μmol/L) and siRNA (40 nmol/L) for 72 hours.

### WB analysis

Primary antibodies for WB analysis against ANXA1 (D5V2T; 1:1,000), LCN2 (D4M8L; 1:1,000), STAT3 (79D7, RRID: AB_331269; 1:2,000), phospho-STAT3 (D3A7; 1:2,000), and GAPDH (14C10, RRID: AB_561053; 1:5,000) were purchased from Cell Signaling Technology, whereas primary antibodies against CLDN4 (EPRR17575; 1:1,000) were purchased from Abcam. Anti-rabbit IgG horseradish peroxidase–conjugated whole antibody (donkey NA934, RRID: AB_772206) was purchased from Cytiva. For WB analysis, cells were harvested, washed in PBS, lysed in radioimmunoprecipitation assay buffer (1% Triton X-100, 0.1% SDS, 50 mmol/L Tris–HCl, pH 7.4, 150 mmol/L NaCl, 1 mmol/L EDTA, 1 mmol/L EGTA, 10 mmol/L β-glycerol-phosphate, 10 mmol/L NaF, and 1 mmol/L sodium orthovanadate), and supplemented with a protease inhibitor cocktail (Roche Applied Sciences cat. #04693132001). Proteins were separated using SDS-PAGE and transferred onto membranes, which were subsequently incubated with the indicated primary and secondary antibodies. Chemiluminescence was detected using an enhanced chemiluminescence reagent (Cytiva, RPN2232). Bands were detected using an Amersham ImageQuant 800 (Cytiva).

### Statistical analysis

Patient characteristics and laboratory findings were assessed using Fisher exact test or the Mann–Whitney U test. Receiver operating characteristic (ROC) curve analyses were performed for NLR, PLR, SII, and PNI. The optimal cutoff value for NLR was determined using the Youden index. The Kaplan–Meier method was used to analyze PFS and OS. PFS and OS were assessed using log-rank tests. Univariate analyses were performed using a logistic regression model to evaluate the factors associated with early resistance to alectinib in *ALK*-positive lung cancer. All patients were included in the analyses. Due to the small sample size, multivariate analyses were performed using a Firth penalized logistic regression (20), which included variables with presumed clinical relevance (PS, brain and liver metastases, and NLR; refs. 11, 21, 22). In the volcano plot, the −log_10_ adjusted *P* value was plotted on the *y*-axis, and the log_2_ fold change was plotted on the *x*-axis. All statistical analyses were performed using STATA version 19.5 (StataCorp; RRID: SCR_012763) and GraphPad Prism 10 (GraphPad Software; RRID: SCR_002798); *P* values < 0.05 were considered statistically significant.

## Results

### Patient characteristics

A total of 103 patients with stage III/IV disease harboring *ALK* fusion–positive NSCLC without an indication for radical radiotherapy or surgery or with *ALK* fusion–positive recurrent NSCLC were consecutively enrolled in this study. The clinical characteristics of the 103 patients are listed in Supplementary Table S1. The median patient age was 65 years (range, 24–89). Of the included patients, 43% were men, 87% had a PS of 0 to 1, 93% had adenocarcinoma, and 54% were nonsmokers. Brain and liver metastases were found in 21% and 17%, respectively, of the overall cohort. The laboratory findings of all the patients are presented in Supplementary Table S2. The median PFS was 28.7 months [95% confidence interval (CI), 17.8–50.2 months; Supplementary Fig. S1A]. The median OS was 80.6 months [95% CI, 55.7 months–not estimable (NE); Supplementary Fig. S1B].

### Clinical and laboratory findings of the early resistance and responder groups

The early resistance group included 18% (19/103), and the responder group included 82% (84/103) of the patients. The clinical characteristics of each group are presented in Table 1. The early resistance group had a significantly higher proportion of brain metastasis (*P* = 0.027). In contrast, there were no significant differences in age, sex, stage, PS, histology, smoking history, or liver metastasis. The laboratory findings for each group are presented in Table 2. Compared with the responder group, the early resistance group had a higher white blood cell count, neutrophil count, NLR, PLR, SII, alanine aminotransferase levels, lactate dehydrogenase levels, and C-reactive protein (CRP) levels and lower lymphocyte count, PNI, total protein levels, and albumin levels.

**Table 1.** Patient characteristics of early resistance and responder groups.

​	Early resistance (*n* = 19)	Responder (*n* = 84)	*P* value
Median age, years (range)	63 (36–79)	65 (24–89)	​
Age (≥65 years/<65 years)	9 (47%)/10 (53%)	43 (51%)/41 (49%)	0.804
Sex (male/female)	8 (42%)/11 (58%)	36 (43%)/48 (57%)	1
Stage (III, IV/recurrent)	17 (89%)/2 (11%)	59 (70%)/25 (30%)	0.146
PS (0–1/2–3)	16 (84%)/3 (16%)	74 (88%)/10 (12%)	0.703
Histology (Ad/others)	17 (89%)/2 (11%)	79 (94%)/5 (6%)	0.610
Smoking history (yes/no)	9 (47%)/10 (53%)	37 (44%)/46 (55%)	1
Metastasis of brain (yes/no)	8 (42%)/11 (58%)	14 (17%)/70 (83%)	0.027
Metastasis of liver (yes/no)	6 (32%)/13 (68%)	11 (13%)/73 (87%)	0.081

Abbreviations: Ad, adenocarcinoma.

**Table 2.** Laboratory findings of early resistance and responder groups.

​	Early resistance median (IQR)	Responder median (IQR)	*P* value	​	Early resistance median (IQR)	Responder median (IQR)	*P* value
WBC, /μL	9,480 (6,600–13,970)	6,620 (5,000–8,200)	0.004	TP, g/dL	6.3 (5.9–6.8)	7 (6.7–7.3)	0.001
Hb, g/dL	11.9 (10.9–13.7)	12.6 (11.5–13)	0.221	Alb, g/dL	2.9 (2.5–3.9)	3.9 (3.5–4.2)	<0.001
Plt, ×10^4^/μL	28.6 (20.9–41.4)	25 (19.7–31.2)	0.269	AST, U/L	22 (19–29)	19 (16–24)	0.050
Neut, /μL	7,074 (4,970–12,125)	4,364 (3,230–5,872)	<0.001	ALT, U/L	23 (14–36)	14 (12–20)	0.020
Lym, /μL	1,022 (866–1,561)	1,474 (1,112–1,734)	0.010	Cre, mg/dL	0.63 (0.53–0.82)	0.67 (0.59–0.80)	0.642
NLR	7.60 (4.29–13.35)	3.07 (2.18–4.20)	<0.001	LDH, U/L	230 (181–419)	194 (165–232)	0.049
PLR	326 (178–393)	175 (133–230)	0.002	CRP, mg/dL	2 (0.31–7.36)	0.15 (0.05–1.32)	<0.001
SII	2,384 (1,091–3,542)	804 (505–1,249)	<0.001	CEA, ng/mL	7.70 (2.64–78.2)	9.40 (2.60–23.2)	0.781
PNI	35.1 (30.1–45.3)	45.8 (42.2–50.2)	<0.001	​	​	​	​

Abbreviations: Alb, albumin; ALT, alanine aminotransferase; AST, aspartate aminotransferase; CEA, carcinoembryonic antigen; Cre, creatinine; Hb, hemoglobin; IQR, Interquartile range; LDH, lactate dehydrogenase; Lym, lymphocyte; Neut, neutrophil; Plt, platelet; TP, total protein; WBC, white blood cell.

**Figure 1.**
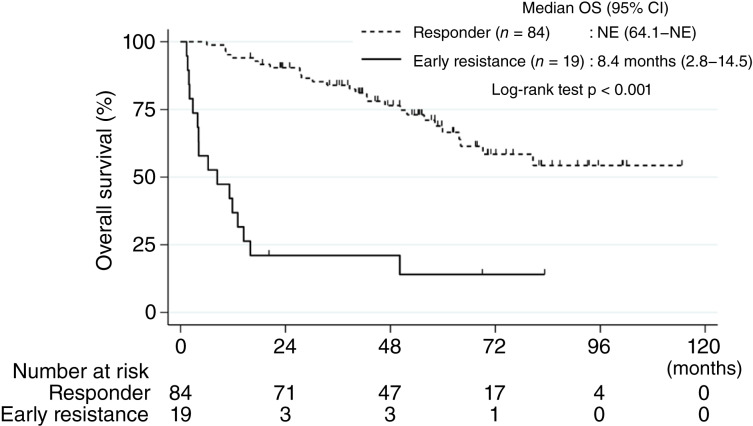
Kaplan–Meier curves for OS. Kaplan–Meier curve showing OS between the early resistance group (solid line) and the responder group (dashed line).

The median PFS was 1.6 months in the early resistance group and 48.1 months in the responder group (*P* < 0.001; Supplementary Fig. S2). The median OS was significantly shorter in the early resistance group than in the responder group (8.4 months vs. NE, *P* < 0.001; Fig. 1).

### Identification of biomarkers of early resistance to alectinib

ROC analysis for the presence of early resistance showed that the areas under the curves for NLR, SII, PNI, and PLR were 0.832, 0.801, 0.775, and 0.720, respectively (Supplementary Fig. S3A–S3D). Therefore, we focused on the NLR. The cutoff point for NLR based on the Youden index was 4.24 (sensitivity, 79%; specificity, 77%). Univariate analyses revealed that brain metastases and an NLR ≥4.24 were significantly associated with increased risks of early resistance [Table 3; brain metastases: Odds ratio (OR) 3.636; 95% CI, 1.239–10.699; *P* = 0.019; NLR: OR 12.434; 95% CI, 3.684–41.965; *P* < 0.001]. Although exploratory due to the limited number of cases, multivariate analyses revealed that an NLR ≥4.24 remained significantly associated with increased risks of early resistance (Supplementary Table S3).

**Table 3.** Univariate analyses of the factors associated with early resistance to alectinib.

​	OR (95% CI)	*P* value
Age (≥65 years vs. <65 years)	0.858 (0.316–2.325)	0.764
Sex (male vs. female)	0.969 (0.353–2.657)	0.952
PS (2–3 vs. 0–1)	1.387 (0.342–5.619)	0.646
Histology (others vs. Ad)	1.858 (0.332–10.396)	0.480
Smoking history (yes vs. no)	1.118 (0.411–3.038)	0.826
Metastasis of brain (yes vs. no)	3.636 (1.239–10.699)	0.019
Metastasis of liver (yes vs. no)	3.062 (0.963–9.736)	0.058
NLR (≥4.24 vs. <4.24)	12.434 (3.684–41.965)	<0.001

Abbreviations: Ad, adenocarcinoma.

### Adverse events

Adverse events ≥ grade 3 were observed in 13 patients (13%; Supplementary Table S4). No significant differences were observed between the early resistance and responder groups. In eight patients with adverse events ≥grade 3, alectinib was withdrawn or the dose was reduced. There was no significant difference in the frequency of dose reduction or treatment discontinuation between the early resistance and responder groups (2 vs. 6, *P* = 1).

### ANXA1, LCN2, and CLDN4 expression in the early resistance group

**Figure 2.**
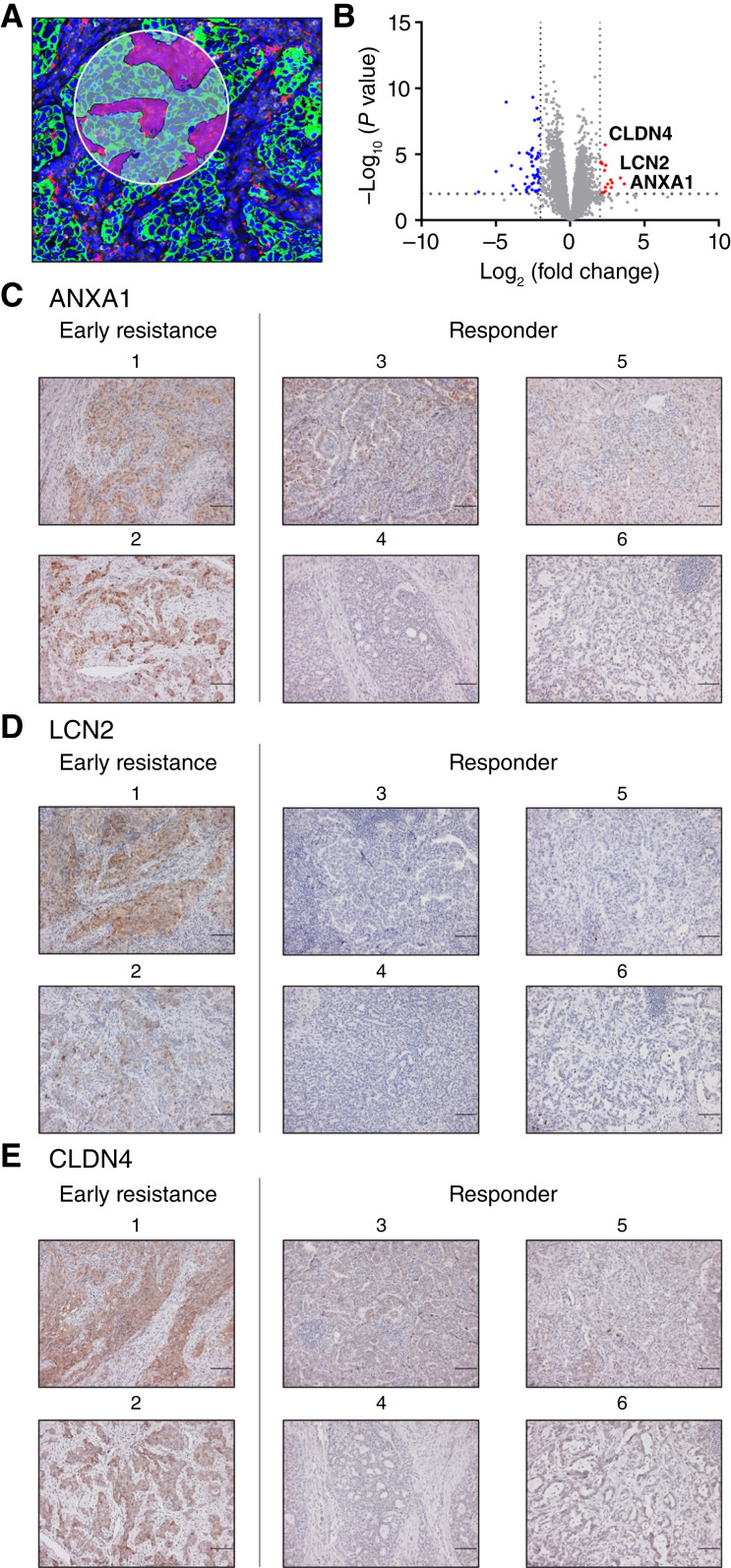
Spatial transcriptome analysis and IHC staining of candidate markers. **A,** Representative image obtained with the GeoMx DSP system. ROIs were defined within the tumor area. Green: pan-cytokeratin, epithelial cell marker; red: CD68, macrophage marker; yellow: CD45, pan-leukocyte cell marker; and blue: SYTO13, nuclear DNA marker. **B,** Volcano plot of the RNA expression that is differentially expressed by early resistance and responder groups in the pan-cytokeratin positive areas of ROIs. **C–E,** Representative images of ANXA1 (**C**), LCN2 (**D**), and CLDN4 **(E**) IHC staining in each patient. Scale bars, 100 μm.

Six surgically resected samples were analyzed using a NanoString GeoMx instrument and IHC. The clinical characteristics of the six patients are shown in Supplementary Table S5. Two patients were in the early resistance group, and four were in the responder group. Thirty-four ROIs were selected that were representative of each tumor (Fig. 2A). Analysis of RNA from tumor regions demonstrated that *ANXA1*, *LCN2*, and *CLDN4* were upregulated in the tumor regions of the early resistance group (Fig. 2B). Similarly, IHC analysis also demonstrated that ANXA1, LCN2, and CLDN4 expression was elevated in the early resistance group (Fig. 2C–E).

**Figure 3.**
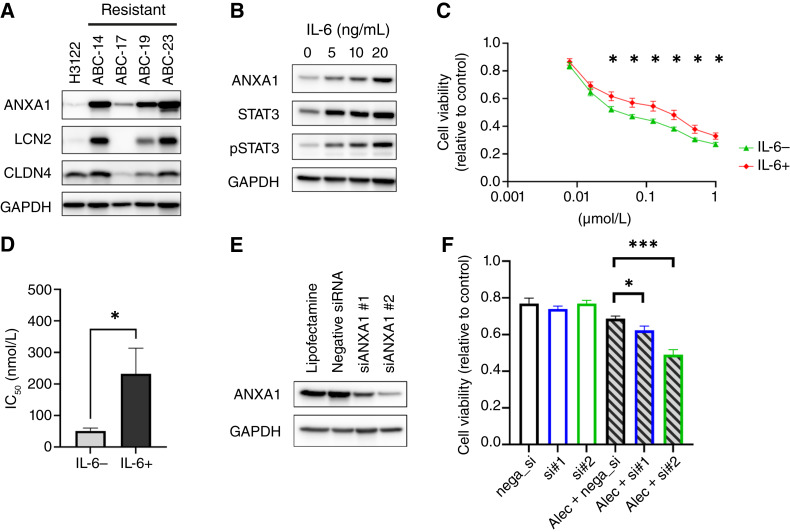
Effects of IL-6 and ANXA1 on alectinib sensitivity. **A,** WB analysis of ANXA1, LCN2, and CLDN4 in H3122, ABC-14, ABC-17, ABC-19, and ABC-23 cells. Data are representative of three independent experiments with similar results. **B,** WB analysis of ANXA1, STAT3, and phospho-STAT3 (pSTAT3) in H3122 cells treated with varying concentrations of IL-6 for 24 hours. Data are representative of three independent experiments with similar results. **C,** Cell proliferation assays at increasing concentrations with or without IL-6 (20 ng/mL) in H3122 cells treated with alectinib for 72 hours. Data are presented as the mean ± standard error (*n* = 9 per group). *, *P* < 0.05, Student *t* test. **D,** IC_50_ values of alectinib in H3122 cells with or without IL-6 treatment (20 ng/mL). Data are presented as the mean ± standard error (*n* = 9 per group). *, *P* < 0.05, Student *t* test. **E,** WB analysis of ANXA1 expression in ABC-14 cells transfected with negative control siRNA or two different siRNAs targeting ANXA1 (siANXA1 #1, #2). Data are representative of three independent experiments with similar results. **F,** Cell proliferation assays in ABC-14 cells transfected with negative control siRNA (nega_si) or different siRNAs targeting ANXA1 [siANXA1 #1 (si#1), #2 (si#2)] treated with alectinib (Alec; 0.1 μmol/L) for 72 hours. *n* = 5 per group. Error bars represent the standard error. *, *P* < 0.05; ***, *P* < 0.001, Student *t* test comparing each siANXA1 group (Alec + si#1 or #2) with the negative control group (Alec + nega_si).

We examined ANXA1, CLDN4, and LCN2 expression in the *ALK*-positive cell lines ABC-14, ABC-17, ABC-19, and ABC-23, which were established from patients with early resistance to alectinib (6, 19), and in H3122, which is sensitive to alectinib. Among the three candidate genes, ANXA1 expression was consistently upregulated in all early resistant cell lines compared with H3122 (Fig. 3A). Therefore, we focused on ANXA1. We next examined the relationship between inflammation and resistance, as inflammatory markers such as CRP and NLR were elevated in the early resistant group. To further explore this, we performed IHC for IL-6 and phospho-STAT3 in the same patients. The expression of IL-6 and phospho-STAT3 tended to be higher in patients with early resistance (Supplementary Fig. S4A and S4B), suggesting that activation of the IL-6/STAT3 pathway may contribute to early resistance. In addition, based on previous findings that IL-6 levels are positively correlated with CRP or NLR (23, 24), H3122 cells were stimulated with recombinant IL-6. IL-6 stimulation led to increased expression of ANXA1 and phospho-STAT3 (Fig. 3B). Furthermore, IL-6 stimulation attenuated the antiproliferative effect of alectinib in H3122 cells (Fig. 3C and D). To further determine the contribution of IL-6/STAT3 signaling, we next examined the effect of ruxolitinib, a selective JAK1/2 inhibitor (25). Cotreatment with ruxolitinib and alectinib attenuated the IL-6–induced ANXA1 upregulation in H3122 cells (Supplementary Fig. S4C). In addition, ruxolitinib partially rescued sensitivity to alectinib (Supplementary Fig. S4D). These findings suggest that STAT3 signaling may contribute to ANXA1 induction and reduced alectinib sensitivity.

Transfection with siRNAs targeting ANXA1 (siANXA1) reduced the expression of ANXA1 in ABC-14 cells (Fig. 3E). The combination of siANXA1 and alectinib significantly suppressed cell proliferation compared with treatment with alectinib alone (Fig. 3F), suggesting that ANXA1 contributes to early resistance to alectinib.

## Discussion

Few observational studies have assessed the clinical and molecular characteristics of patients with *ALK*-positive lung cancer who exhibit early resistance to alectinib. In the current study, a subset of patients with *ALK*-positive NSCLC who experienced early resistance to alectinib exhibited distinct clinical and molecular characteristics. Consistent with previous reports (4, 5), alectinib demonstrated excellent PFS in our study. However, early resistance to alectinib occurred in 18% of the study cohort. The early resistance group also had a significantly shorter OS than the responder group. Therefore, the risk factors for early resistance need to be identified, and different treatment options need to be considered.

In our study, brain metastasis was a clinical predictor of early resistance. A previous study reported that patients on first-line ALK-TKI therapy with brain metastases had a significantly increased risk of progression, although crizotinib was mainly used as the first-line treatment (26). Although alectinib has demonstrated favorable intracranial activity compared with crizotinib (27, 28), our findings suggest that more intensive treatment may be needed in patients with brain metastases. Such patients may also warrant closer clinical attention, including more careful assessment of the central nervous system. In a meta-analysis, lorlatinib, a third-generation ALK-TKI, was the most effective in prolonging PFS in patients with central nervous system metastasis (29). Moreover, the CROWN trial demonstrated that lorlatinib significantly improved systemic and intracranial PFS compared with crizotinib as a first-line treatment, although with higher toxicity (30). These data suggest that lorlatinib may be a more suitable first-line option in patients with brain metastases. Furthermore, patients with high NLR who were at increased risk of early resistance in our study may also warrant consideration of first-line lorlatinib. However, further investigation is required to clarify the efficacy of this approach in this subgroup. As another potential strategy, combination therapy with alectinib and bevacizumab has shown promising efficacy in early-phase studies involving patients with brain metastases (31, 32). Inflammation-associated signaling can enhance vascular endothelial growth factor (VEGF) expression and angiogenesis (33), and VEGF-driven pathways have been implicated in tumor survival and therapeutic resistance (34). Therefore, anti-VEGF agents may also help mitigate inflammation-driven early resistance to alectinib. These treatments may be considered first-line options for *ALK*-positive patients at risk of early resistance.

Neutrophils are involved in tumor promotion by producing cytokines and proteases (35). The NLR is a systemic inflammatory marker considered a prognostic factor for NSCLC (36, 37). A retrospective cohort study revealed that in *ALK*-positive lung cancer, both baseline NLR and its change after alectinib treatment were associated with clinical outcomes (11). Our results suggest that this may be a risk factor for early resistance. Furthermore, high NLR and brain metastases were significantly more frequent in the early resistance group in our study, consistent with previous studies suggesting an association between elevated NLR and brain metastases in NSCLC (38). In addition, compared with the responder group, the early resistance group had lower nutritional status. Serum albumin is used to assess nutritional status and provides useful prognostic significance in various cancers, including NSCLC (39). The PNI is a nutritional and immunologic index and has been reported to be an independent prognostic factor for lung cancer (40). Previous studies have shown that the PNI is correlated with treatment response to crizotinib (41) and PFS with alectinib (9). In our study, the albumin and PNI levels were significantly lower in the early resistance group. These findings suggest that systemic inflammation and poor nutritional status may contribute to early resistance to alectinib.

At the molecular level, the expression of ANXA1, LCN2, and CLDN4 was upregulated at both the RNA and protein levels in the early resistance group. Among these, ANXA1 was consistently upregulated in early resistant cell lines, and functional experiments demonstrated its role in mediating resistance to alectinib. ANXA1—a member of the annexin superfamily of calcium-dependent phospholipid-binding proteins—was elevated in various cancers (42) and contributes to the growth and invasion of lung cancer cells (43). Overexpression of ANXA1 is associated with cisplatin resistance (44) and reduces sensitivity to osimertinib, an epidermal growth factor receptor (EGFR)-TKI, with knockdown restoring sensitivity in *EGFR*-mutated lung cancer cells (45). Our results were consistent with the hypothesis that ANXA1 contributes to drug resistance in cancer cells. In addition, previous studies have shown that ANXA1 can activate oncogenic pathways such as the MAPK/ERK pathway (46) and the PI3K/Akt pathway (47), suggesting that ANXA1 overexpression may sustain survival signaling independently of ALK inhibition. Although further validation is required, such a mechanism could potentially contribute to early resistance to alectinib. Whereas the roles of LCN2 and CLDN4 in *ALK*-positive lung cancer remain unclear, LCN2, a member of the lipocalin family (48), has been associated with chemoresistance in colorectal cancer (49), whereas CLDN4, a tight junction protein (50), may limit drug penetration into tumors and has been shown to affect chemosensitivity in breast cancer (51, 52). Further studies are needed to determine whether these molecules contribute to the early resistance to alectinib.

Given the higher NLR and elevated CRP levels in the early resistance group, we explored the potential contribution of inflammation to resistance. Previous studies have reported that systemic inflammatory markers such as NLR and CRP levels are positively correlated with IL-6 (23, 53). In our *in vitro* experiments, IL-6 stimulation increased ANXA1 expression and STAT3 activation. These findings are consistent with those of previous reports showing that the IL-6/STAT3 signaling pathway correlates with ANXA1 expression in several cancer types (54–56). Moreover, IL-6 stimulation reduced the sensitivity of cancer cells to alectinib, suggesting that inflammation-induced upregulation of ANXA1 contributes to early resistance to alectinib. In addition, knockdown of ANXA1 restored alectinib sensitivity in early resistant cell lines. Consistent with this mechanism, JAK1/2 inhibition with ruxolitinib attenuated IL-6-induced ANXA1 upregulation and partially restored alectinib sensitivity *in vitro*. Consequently, therapeutic strategies targeting IL-6–mediated inflammatory signaling, including the potential use of anti–IL-6 agents in combination with alectinib, may help mitigate inflammation-driven early resistance. Furthermore, MDX-124, a humanized monoclonal antibody targeting ANXA1, suppresses tumor growth in ANXA1-overexpressing tumors (57) and is currently under clinical development (58). We suggest that ANXA1-targeted therapies, such as MDX-124, represent a promising treatment strategy for patients with *ALK*-positive lung cancer with early resistance to alectinib. Future preclinical studies using MDX-124 would be particularly valuable for determining whether direct inhibition of ANXA1 can reverse or prevent early resistance to alectinib. Supplementary Figure S5 summarizes this proposed mechanism.

Our study had some limitations. First, *ALK* variants were not evaluated. Second, because this was a retrospective observational study, reverse causation cannot be excluded; early resistance itself may elevate systemic inflammatory markers such as NLR or CRP. Therefore, the associations observed in this study should be interpreted as correlational rather than causal, and residual confounding may still remain despite adjustment for available clinical factors. Third, the limited sample size restricted our ability to perform a detailed evaluation of multiple inflammatory markers. In addition, only an exploratory multivariate analysis using Firth penalized logistic regression could be performed, and the results should be interpreted with caution. Validation in a larger cohort is warranted. Furthermore, the optimal cutoff value for NLR was determined using the Youden index without internal validation such as bootstrap resampling or cross-validation. Therefore, this cutoff should be regarded as exploratory and requires validation in future studies. Fourth, the number of cases analyzed using GeoMx was limited because of the difficulty in obtaining sufficient quality for ROI selection. Fifth, treatment patterns after alectinib resistance could not be thoroughly analyzed due to the limited number of eligible cases. However, our study provided valuable real-world data on early resistance to alectinib.

In conclusion, 18% of patients exhibited early resistance to alectinib, and these patients had a significantly shorter OS. The presence of brain metastases or elevated levels of inflammatory markers, such as NLR, was associated with early resistance to alectinib. Elevated ANXA1 expression, identified through spatial transcriptomics and functional analysis, may contribute to early resistance to alectinib. ANXA1-targeted therapy may overcome the early resistance to alectinib. Further *in vivo* and large-scale clinical studies using blood samples and tumor tissues are required to clarify the clinical relevance of the IL-6–STAT3–ANXA1 pathway. In addition, continued investigations, including the development of additional biomarkers in the early resistance group, exploration of alternative ALK inhibitors, and optimal treatment sequencing, are necessary.

## Supplementary Material

Figure S1 Kaplan–Meier curves of progression-free survival (PFS) and overall survival (OS) in all patients.

Figure S2 Kaplan–Meier curves for progression-free survival (PFS).

Figure S3 Receiver operating characteristic (ROC) curves.

Figure S4 Immunohistochemical staining and cell proliferation assay.

Figure S5 Proposed schematic model of early resistance to alectinib mediated by inflammation.

Supplementary Table Supplementary Table1-5

## Data Availability

The data generated in this study are available upon request from the corresponding author. The GeoMx data analyzed in this study are available in the NCBI Gene Expression Omnibus repository (GSE309894).
