# Supplementary figures and images for: Clinical Characteristics and Spatial Transcriptome Analysis of Non–Small Cell Lung Cancers Exhibiting Early Alectinib Resistance: A Retrospective OLCSG Study
Source: Cancer Res Commun. 2026 Feb 6;6(2):284–93. doi: 10.1158/2767-9764.CRC-25-0545 (PMC12877432; doi:10.1158/2767-9764.CRC-25-0545)

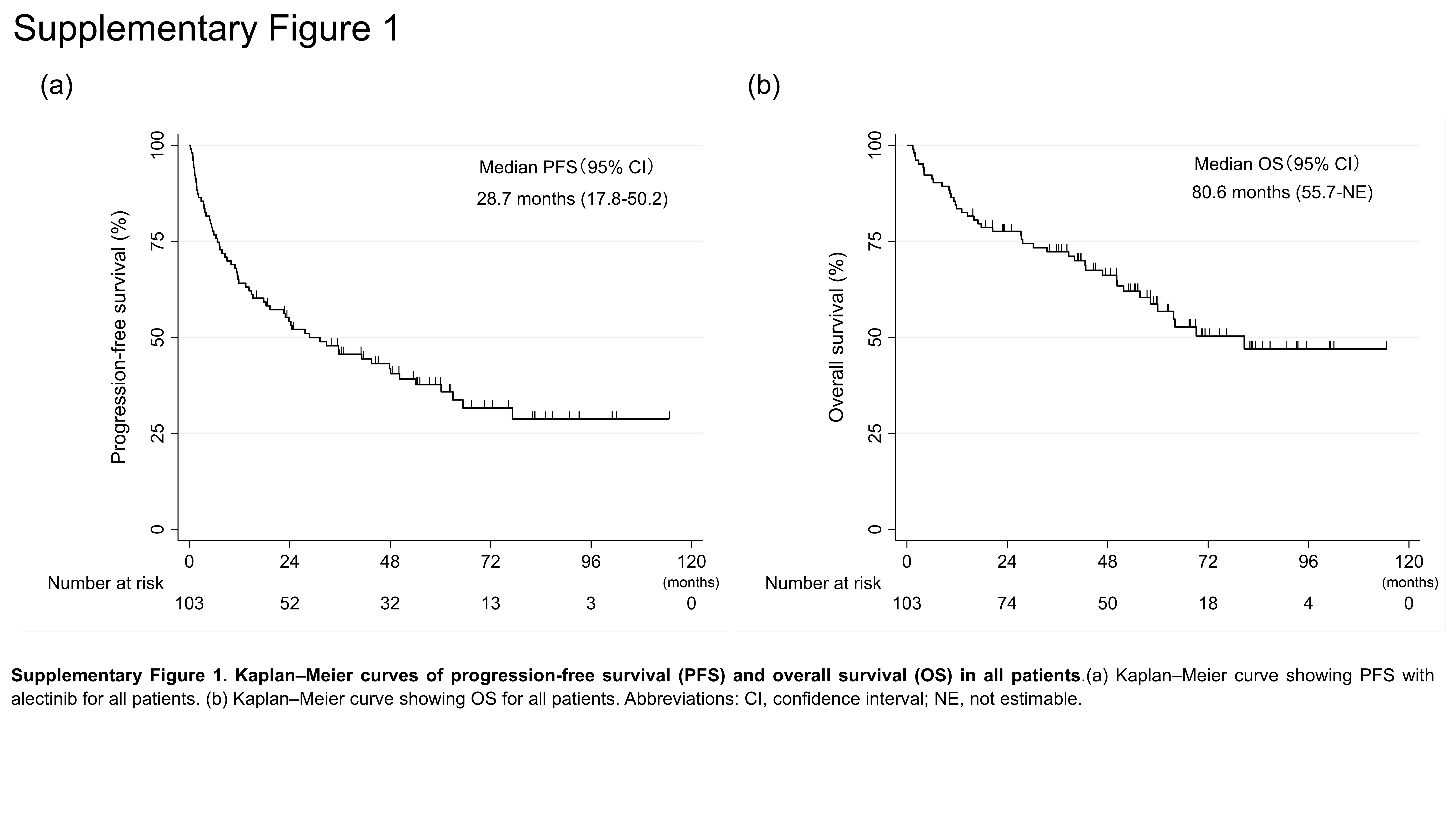

Supplement: Figure S1 — Kaplan–Meier curves of progression-free survival (PFS) and overall survival (OS) in all patients. [file crc-25-0545_figure_s1_suppsf1.png]

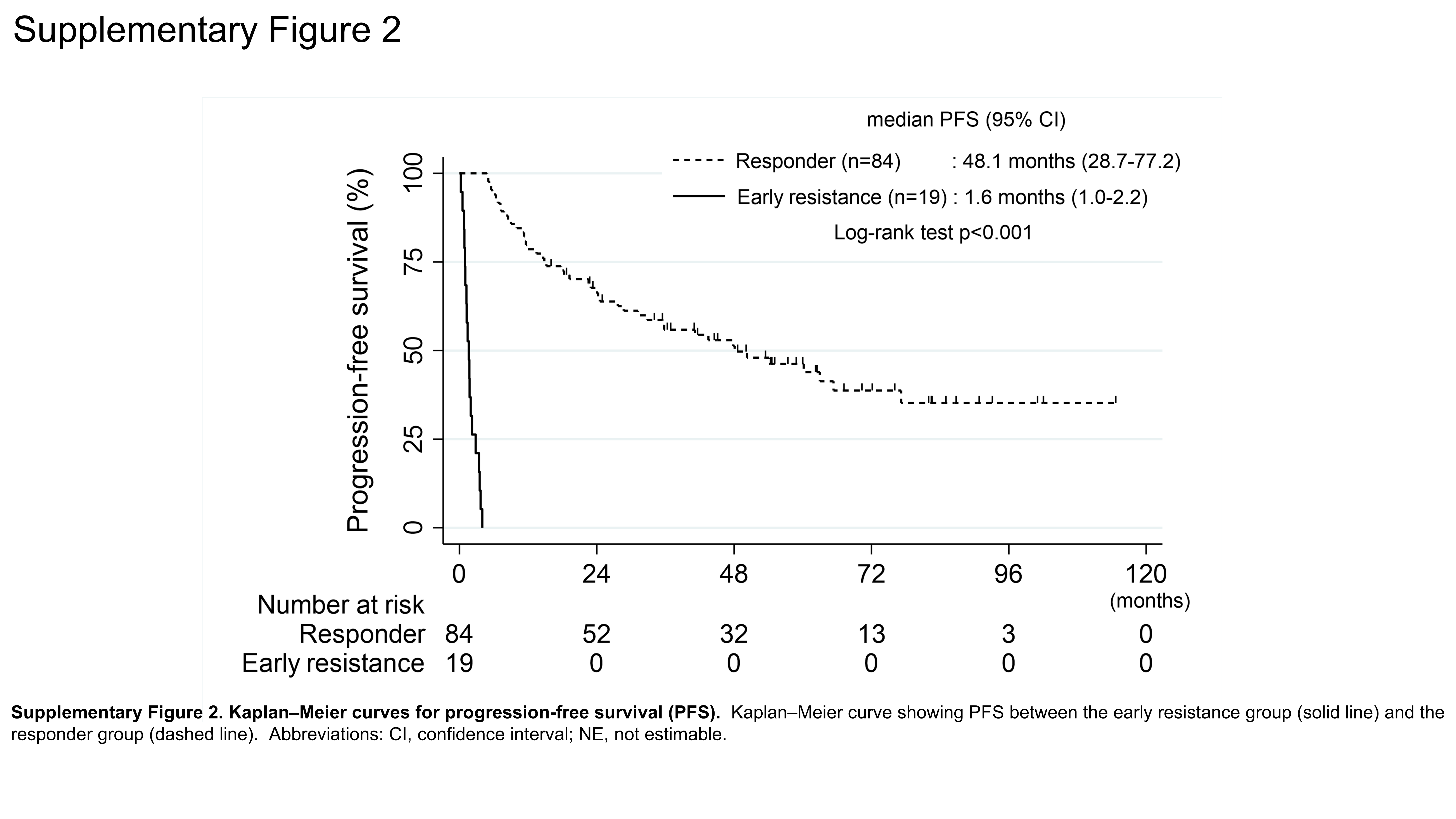

Supplement: Figure S2 — Kaplan–Meier curves for progression-free survival (PFS). [file crc-25-0545_figure_s2_suppsf2.png]

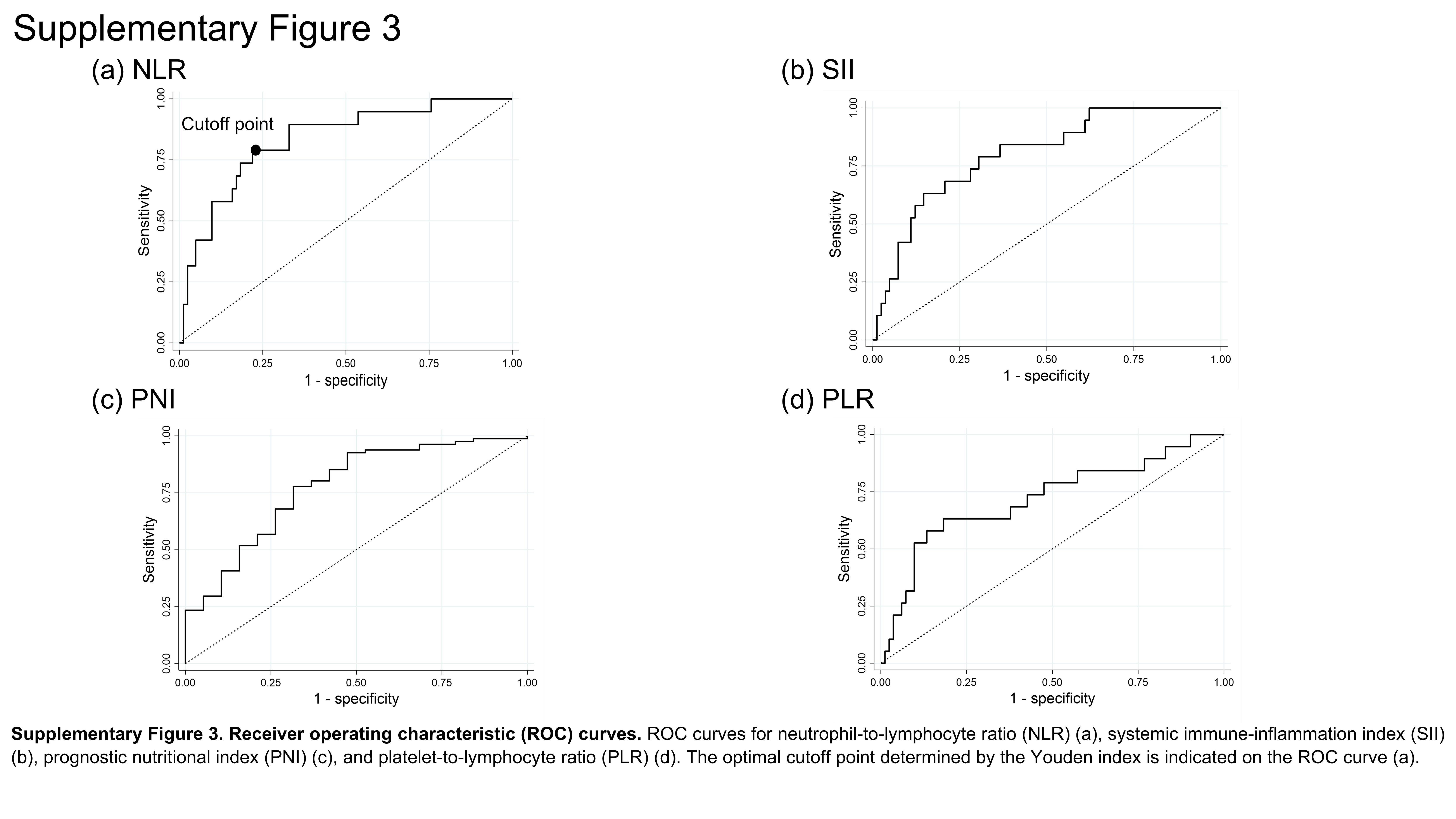

Supplement: Figure S3 — Receiver operating characteristic (ROC) curves. [file crc-25-0545_figure_s3_suppsf3.png]

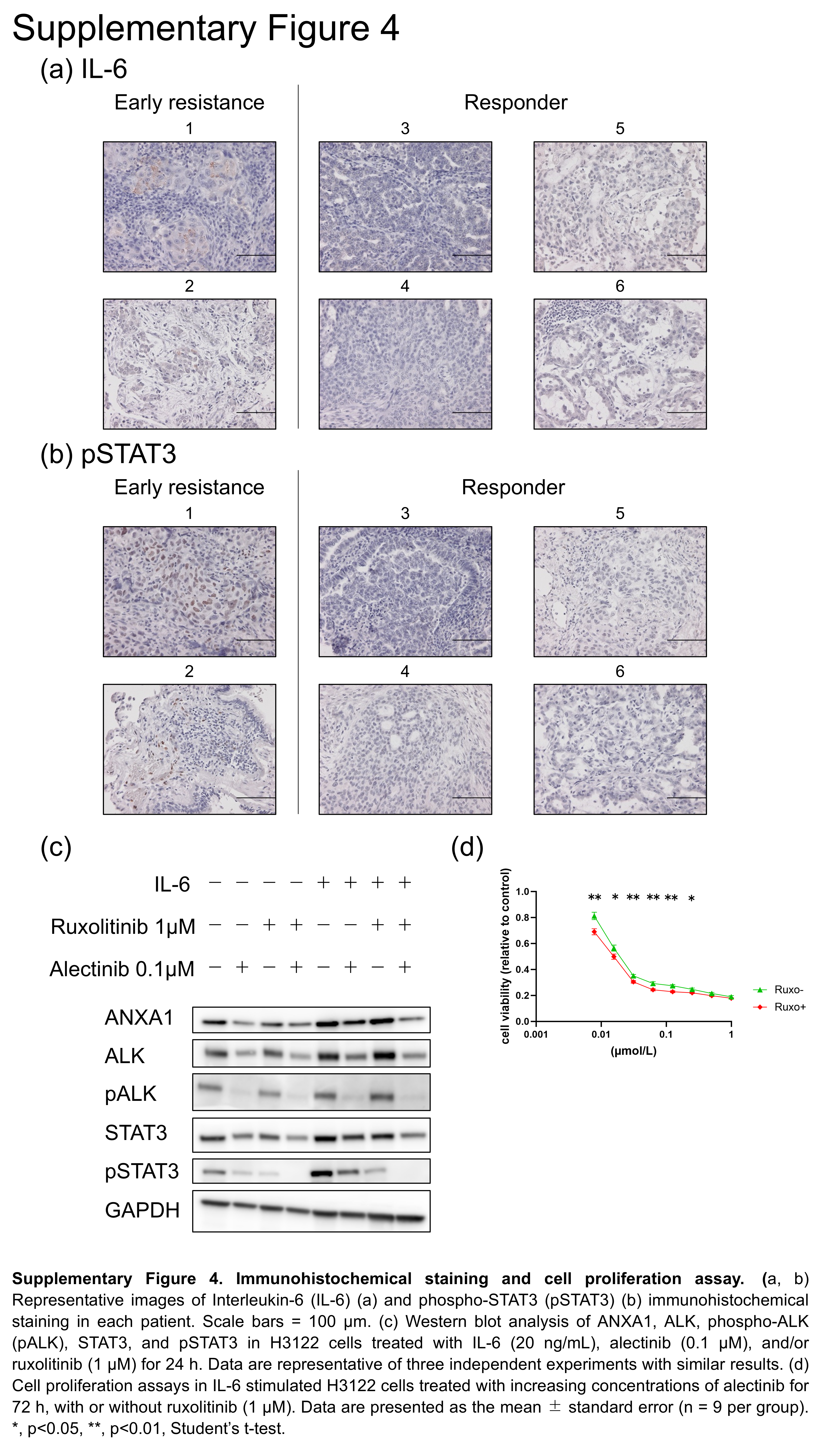

Supplement: Figure S4 — Immunohistochemical staining and cell proliferation assay. [file crc-25-0545_figure_s4_suppsf4.png]

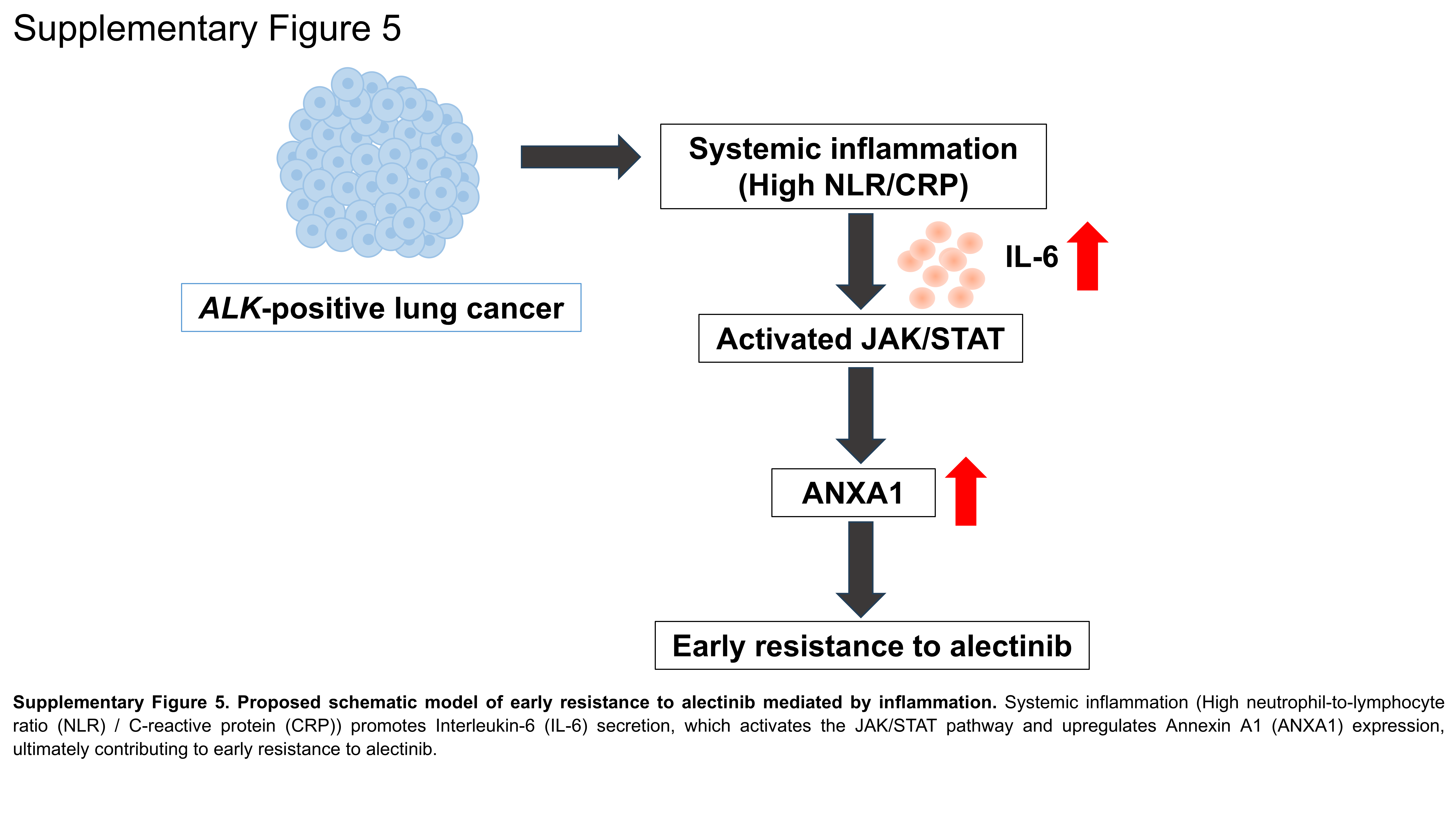

Supplement: Figure S5 — Proposed schematic model of early resistance to alectinib mediated by inflammation. [file crc-25-0545_figure_s5_suppsf5.png]
