# Supplementary material for: Clinical Characteristics and Spatial Transcriptome Analysis of Non–Small Cell Lung Cancers Exhibiting Early Alectinib Resistance: A Retrospective OLCSG Study
Source: Cancer Res Commun. 2026 Feb 6;6(2):284–93. doi: 10.1158/2767-9764.CRC-25-0545 (PMC12877432; doi:10.1158/2767-9764.CRC-25-0545)
Supplement: Supplementary Table — 1-5 [file crc-25-0545_supplementary_table_suppst.docx]

| Supplementary Table 1 Patient characteristics (n=103) | |
| --- | --- |
| Median age, years (range) | 65 (24-89) |
| Sex (male/female) | 44 (43%)/59 (57%) |
| Stage (Ⅲ, Ⅳ/recurrent) | 76 (74%)/27 (26%) |
| PS (0-1/2-3) | 90 (87%)/13 (13%) |
| Histology (Ad/others) | 96 (93%)/7 (7%) |
| Smoking history (never/former/current/unknown) | 56 (54%)/31 (30%)/15 (15%)/1 (1%) |
| Metastasis of brain (yes/no) | 22 (21%)/81 (79%) |
| Metastasis of liver (yes/no) | 17 (17%)/86 (83%) |
| Abbreviations: PS, performance status; Ad, adenocarcinoma | |

| Supplementary Table 2 Laboratory findings of all Patients | | | |
| --- | --- | --- | --- |
| WBC, /µL (range) | 6700 (2279-33510) | **TP, g/dL (range)** | 6.9 (4.9-8.2) |
| Hb, g/dL (range) | 12.6 (6.7-16.8) | **Alb, g/dL (range)** | 3.9 (1.7-5.2) |
| Plt, x10^4^/µL (range) | 25.1 (11.0-64.7) | **AST, U/L (range)** | 19 (10-92) |
| Neut, /µL (range) | 4739 (1585-26808) | **ALT, U/L (range)** | 16 (5-160) |
| Lym, /µL (range) | 1449 (91-3304) | **Cre, mg/dL (range)** | 0.67 (0.42-7.06) |
| NLR, (range) | 3.36 (0.87-93.6) | **LDH, U/L (range)** | 199 (110-924) |
| PLR, (range) | 182.8 (63.5-5947.7) | **CRP, mg/dL (range)** | 0.23 (0-24.43) |
| SII, (range) | 904.0 (117.3-51081.3) | **CEA, ng/mL (range)** | 9.4 (0.7-1565.1) |
| PNI, (range) | 45.4 (20.5-58.0) |  |  |
| Abbreviations: WBC, white blood cell; Hb, hemoglobin; Plt, platelet; Neut, neutrophil; Lym, lymphocyte; NLR, neutrophil-to-lymphocyte ratio; PLR, platelet-to-lymphocyte ratio; SII, systemic immune-inflammation index; PNI, prognostic nutritional index; TP, total protein; Alb, albumin; AST, aspartate aminotransferase; ALT, alanine aminotransferase; Cre, creatinine; LDH, lactate dehydrogenase; CRP, C-reactive protein; CEA, carcinoembryonic antigen | | | |

| Supplementary Table 3 Multivariate analyses of the factors associated with early resistance to alectinib | | |
| --- | --- | --- |
|  | **Odds Ratio (95% CI)** | **p value** |
| PS (2-3 vs. 0-1) | 0.889 (0.188-4.187) | 0.882 |
| Metastasis of brain (yes vs. no) | 2.236 (0.657-7.606) | 0.197 |
| Metastasis of liver (yes vs. no) | 2.568 (0.676-9.745) | 0.166 |
| NLR (≥4.24 vs. <4.24) | 9.611 (2.922-31.610) | <0.001 |
| Abbreviations: CI, confidence interval; PS, performance status; NLR, neutrophil-to-lymphocyte ratio | | |

| Supplementary Table 4 Adverse events ≥ Grade 3 | | | |
| --- | --- | --- | --- |
|  | **Early resistance (n=19)** | **Responder (n=84)** | **p value** |
| Total patients | 4 (21%) | 9 (11%) | 0.256 |
| Hepatic dysfunction | 1 | 3 |  |
| Anemia | 1 | 1 |  |
| Bronchial infection, pneumonia | 1 | 1 |  |
| Interstitial lung disease | 1 | 0 |  |
| Disseminated intravascular coagulation | 1 | 0 |  |
| Pulmonary embolism | 0 | 1 |  |
| Skin rash | 0 | 1 |  |
| Thrombophlebitis | 0 | 1 |  |
| Renal dysfunction | 0 | 1 |  |
| Edema | 0 | 1 |  |

| Supplementary Table 5 Characteristics of patients | | | | | | | | | | | | | | | | |
| --- | --- | --- | --- | --- | --- | --- | --- | --- | --- | --- | --- | --- | --- | --- | --- | --- |
| Case | Group | age | sex | PS | Histology | Stage | Smoking | Brain-meta | Liver-meta | NLR | PFS  (mos) | OS  (mos) | response | ALK-TKI after Alec | Specimen | ROI |
| 1 | Early-resistance | 42 | F | 1 | Ad | IVB | never | - | - | High | 1 | 81 | PD | Crizo | Lym | 6 |
| 2 | Early-resistance | 75 | M | 0 | Ad | Rec | former | - | + | Low | 3 | 19 | NE | Lorla, Alec | Lung | 7 |
| 3 | Responder | 76 | F | 1 | Ad | IVA | never | - | - | Low | 56 | 56 | NE | - | Lung | 6 |
| 4 | Responder | 53 | F | 0 | Ad | Rec | never | - | - | Low | 64 | 66 | PR | - | Abd | 4 |
| 5 | Responder | 66 | F | 0 | Ad | Rec | never | - | + | Low | 40 | 40 | CR | - | Lung | 4 |
| 6 | Responder | 69 | M | 0 | Ad | Rec | former | - | + | Low | 76 | 99 | CR | Briga | Lym | 7 |
| Abbreviations: F, female; M, male; PS, performance status; Ad, adenocarcinoma; Rec, recurrence; NLR, neutrophil-to-lymphocyte ratio; PFS, progression-free survival; OS, overall survival; mos, months; PD, progressive disease; NE, not evaluable; PR, partial response; CR, complete response; ALK-TKI, anaplastic lymphoma kinase-tyrosine kinase inhibitor, Alec, alectinib; Crizo, crizotinib; Lorla, Lorlatinib; Briga, Brigatinib; Lym, lymph node; Abd, abdominal wall; ROI, region of interest | | | | | | | | | | | | | | | | |
